# Food Insecurity and Bullying Victimization Among 170,618 Adolescents in 59 Countries

**DOI:** 10.3389/fpsyt.2021.766804

**Published:** 2021-11-11

**Authors:** Kaixin Liang, Xinli Chi, Si-Tong Chen, Cain Craig Truman Clark, Yanjie Zhang, Jian Wang

**Affiliations:** ^1^School of Psychology, Shenzhen University, Shenzhen, China; ^2^Institute for Health and Sport, Victoria University, Melbourne, VIC, Australia; ^3^Faculty of Health and Life Sciences, Coventry University, Coventry, United Kingdom; ^4^Health and Exercise Science Laboratory, Institute of Sports Science, Seoul National University, Seoul, South Korea; ^5^Physical Education Unit, School of Humanities and Social Science, The Chinese University of Hong Kong, Shenzhen, China; ^6^School of Public Policy and Management, Anhui Jianzhu University, Hefei, China; ^7^Urban Management Research Center, Anhui Jianzhu University, Hefei, China; ^8^Department of Psychology, Anhui Normal University, Wuhu, China

**Keywords:** food insecurity, bully victimization, adolescents, Global School-based Student Health Survey, meta-analysis

## Abstract

**Background:** Bullying victimization can impose long-lasting impacts on adolescent's current and subsequent well-being. Understanding the correlates of bullying victimization and how to prevent its occurrence is an urgent need. Food insecurity, an indicator of low socioeconomic status, may be related to bullying victimization. However, research on the association between food insecurity and bullying victimization is limited. Using a representative global sample, this study aimed to investigate the association between food insecurity and bullying victimization in adolescents and whether the association varied between country income levels, sexes, and age groups.

**Methods:** Using cross-sectional, school-based data by 170,618 adolescents in 59 countries from the Global School-based Student Health Survey, multivariable logistic regression and meta-analysis were conducted to investigate the overall, country-stratified, sex-stratified, and age-stratified associations.

**Results:** The prevalence of bullying victimization was 33.3%. In the total sample, adolescents with food insecurity showed significantly higher odds for bullying victimization than those without no food insecurity with a pooled odds ratio (OR) being 1.37 (1.28, 1.47). Further, the association was stronger in higher-income countries, females, and older adolescents.

**Conclusions:** Bullying victimization is prevalent among global adolescents with food insecurity being a significant correlate. The identification of adolescents with perceptions of food insecurity and remedying this condition may be important to reduce the prevalence of bullying. This highlights the need to design and implement sex- and age-specific interventions focusing on remedying food insecurity and bullying victimization among in-school adolescents by taking country income levels into account.

## Introduction

Bullying victimization can be defined as the experience of any intentional physical (e.g., hitting), verbal (e.g., abusive), and psychological aggression from peers, which are repeated and intended to cause harm (1). Increasing evidence indicates that being bullied is a common phenomenon during childhood and adolescence. Recently, a global survey revealed that the estimated prevalence of bullying victimization was 30.5%, with the highest proportion being 45.1% in the Eastern Mediterranean region (2). Besides, data from 11 European countries indicated that almost 30% of adolescents are bullied by their peers (3), whilst 37% of American adolescents reported being bullied (4). Evidence from South Asia suggested that 41 to 53% of students experienced bullying (5–7), and a high prevalence of being bullied was also found in Chinese adolescents, where 35.6% and 31.4% of samples experienced traditional bullying and cyberbullying, respectively (8). The risks of being bullied have been well documented, including aggression (9), violence-related behaviors (10), worse academic performance (11, 12), substance misuse (e.g., problematic alcohol use) (11, 13), disorder eating behaviors, depressive symptoms (11, 13), and even suicide ideation (14, 15). Furthermore, being bullied during childhood and adolescence can have long-lasting impacts on later life (16, 17). Owing to the harms of bullying victimization in adolescents, it is important to prevent the occurrence of bullying victimization in adolescents.

A prior and necessary step to preventing bullying victimization is understanding its correlates. Some theories and conceptual frameworks have been applied or proposed to elucidate the dynamics or organize various factors of bullying involvement in recent years. Based on these theories, socioeconomic status (SES) may be an important factor in explaining bullying. Specifically, grounded in general strain theory, experiencing economic problems was identified as a strong signal of bullying by social big data analysis (18). According to social disorganization theory, environments with a high concentration of poverty are more likely to experience disorganization and may increase the risk of developing attitudes and behaviors that are related to bullying (19). Similarly, based on socio-ecological theory, socioeconomic status may play a role in bullying (20). Regarding social capital theory, it can be assumed that low social status (e.g., low SES) serves to maintain bullying victimization (21). Despite the theories mentioned above focusing on the occurrence of bullying perpetration (rather than bullying victimization), some empirical studies have also documented the association between low SES or poverty and bullying victimization. However, effect sizes reported in these studies vary greatly, with some studies reporting moderate to strong associations (22, 23) whilst others reporting weak ones (24). To determine more precisely the exact nature and strength of the relationship between SES and bullying, Tippett and Wolke conducted the first systematic review and meta-analysis and concluded that poverty or low SES was associated with a higher probability of bully victimization (25). Besides, they pointed out that the strength of association between SES and bullying might differ between SES indicators. Different indicators of SES assess distinct aspects of social status and thus may influence adolescent's development in unique ways. Besides, some studies show that subjective SES (i.e., individual perceptions) was more predictive of adverse health outcomes than objective SES (e.g., household income, parental education) (26–28). However, in existing studies investigating the association between SES and bullying, SES was mainly indicated by household income or parental education (25). This may have failed to identify families experiencing the most extreme forms of low SES involving material deprivation (consistently unable to pay for basic necessities), because, for families with the same income level, there may be remarkable variation in access to these resources due to differences in family size, lifestyle, residence, etc.

Food insecurity refers to the lack of adequate nutrition and safe food, or the inability to obtain food in a socially acceptable manner (e.g., resorting to emergency food supply and stealing food) (29). Food insecurity and low SES, despite being discrete constructs, are closely related. Low SES can result in inadequate access to food, and thus subsequently lead to food insecurity (30). Hence, individual perceptions of food insecurity or hunger often coexist with low SES (31) and could be regarded as a subjective measure of SES. Besides, as aforementioned, low SES is a multi-dimensional phenomenon while food insecurity can be viewed as one marker that can capture material deprivation resulting from extreme low SES. Indeed, studies revealed that food insecurity could add additional independent influence on children and adolescent's health outcomes above and beyond the detrimental impact of low SES (32, 33). Long-term food insecurity or perpetuated hunger has adverse impacts on individual's physical and mental health, leading to undesirable developmental outcomes in adolescents, including nutritional deficiencies, obesity, poor academic performance, and even mental disorders (e.g., depression and anxiety) (33–35). These adverse outcomes would further pose adolescents at a high risk of being bullied as those being too distinct from peers (e.g., too short, too tall, too heavy, too skinny, or not smart enough) often become targets for bullying victims (21). With the potential pathway from food insecurity to bullying victimization, food insecurity may be a factor that might underpin bullying victimization.

Indeed, some studies have empirically examined the connection between food insecurity and bullying, but the results are inconsistent and far from consensual. A previous study with a representative US sample found that food-insecure students were more likely to be victims of bullying than their counterparts (36). Another study using data from the School-based Student Health Survey (GSHS) indicated that Algerian adolescents who experienced food insecurity were more likely to report suffering from bullying victimization compared with their counterparts (37). Of note, also using data from the GSHS, research on adolescents in Ghana revealed limited or no association between food insecurity and bully victimization (38). Part of the reason for these inconsistent results may be that the study samples were from different countries. Previous research found that the association between food insecurity and suicide attempts was stronger in countries with a lower prevalence of food insecurity (i.e., higher-income countries) (39), which suggests that the impact of food insecurity may vary between country income levels. Besides, previous research has shown that sex and age were significant correlates of food insecurity and bullying victimization (40). However, the moderating effects of sex and age on the association between SES or food insecurity and bullying remain unclear given the small number of studies on this topic. Therefore, based on the representative multi-country data from the GSHS, this study aimed to explore the association between food insecurity and bullying victimization among adolescents globally and to examine whether the association varies between country income levels, sexes, and age groups.

## Methods

### Study Survey

The GSHS was implemented to identify the risks and protective factors of major non-communicable diseases among school-aged adolescents. Participating countries selected core modules from GSHS Questionnaire addressing the leading causes of morbidity and mortality among children and adults worldwide (e.g., dietary behaviors, physical activity, mental health) in their country-specific questionnaire. GSHS uses a standardized two-stage probability design for the sample selection process within each participating country. Data selection was performed at the unit of a regular class. Sampling bias was considered by adding weight, stratum, and PSU to every student record in a GSHS data file in the weighting process. All three variables are required to be used when analyzing GSHS data to appropriately represent the weighting process and the 2-stage sample design. Weighting accounts for the probability of selection of schools and classrooms and non-responding schools and students, and distribution of the population by grade and sex. Weighting allows GSHS results to be generalized to the entire population of students, not just those who took the survey. All GSHS surveys, as conducted in each respective country, were approved by institutional ethical reviews. Student's participation was fully anonymous and voluntary, and informed consent was sought and obtained, as appropriate, from the students, parents, and/or school officials. Further information can be accessed at https://extranet.who.int/ncdsmicrodata/index.php/catalog/GSHS and https://www.cdc.gov/gshs/index.htm.

Public-use data from 92 countries collected between 2003 and 2017 were available when we conducted the secondary data analysis in March 2021. After checking whether there were variables required for this study, we finally included 59 countries (33 countries were excluded for not including the variables required). One of the purposes of the GSHS is to establish trends in the prevalence of health behaviors and protective factors for evaluating school health and youth health promotion. For this purpose, some countries would conduct or have conducted GSHS more than once to monitor the development trend of youth health behaviors while other countries only conducted once limited by insufficient funds, staff turnover, or other in-country barriers. If there were more than two datasets from the same country, we selected the most recent dataset. There were 181,912 adolescents in the 59 included countries. After excluding data of 11,294 participants reporting 11 years or younger and 18 years or older, a total of 170,618 adolescents in 59 countries (7 low-income countries, 24 lower-middle-income countries, 16 upper-middle-income countries, and 12 high-income countries) consist of the final analytical sample (no missing data). The sample characteristics are provided in Table 1.

**Table 1:** Prevalence of food insecurity and bullying victimization by countries, sexes, and age groups.

			**Sample size**	**Food insecurity (%)**	***p-*value**	**Bully victimization (%)**	***p-*value**
Income level	Low-income countries	Afghanistan	1,515	21.0	<0.001	41.0	<0.001
		Kenya	2,239	12.7		54.5	
		Liberia	831	10.9		46.4	
		Mozambique	1,018	10.5		42.2	
		Nepal	5,333	4.0		50.6	
		Tanzania	1,572	3.4		23.3	
		Uganda	2,352	8.4		43.6	
		*Subtotal*	1,4860	8.3		48.5	
	Lower middle-income countries	Bangladesh	2,647	13.0		24.0	
		Belize	1,391	14.1		44.3	
		Bolivia	3,061	8.2		30.6	
		Ecuador	1,673	4.2		25.9	
		Egypt	1,942	3.8		68.6	
		Ghana	1,138	13.7		60.6	
		Guyana	2,031	7.1		35.9	
		Honduras	1,484	3.9		30.6	
		Indonesia	9,451	3.8		20.2	
		Jordan	1,592	12.3		39.0	
		Lao	3,273	1.2		11.5	
		Mauritania	1,559	9.6		43.5	
		Mongolia	4,717	1.5		27.5	
		Morocco	4,717	8.3		36.2	
		Pakistan	4,294	5.1		40.8	
		Philippines	7,580	7.1		48.6	
		Solomon Islands	1,039	10.1		64.3	
		Sri Lanka	3,063	2.8		37.8	
		Timor-Leste	2,234	10.4		26.0	
		Tonga	2,502	10.3		36.9	
		Tunisia	2,161	7.7		30.4	
		Vanuatu	1,673	7.9		49.2	
		Viet Nam	2,792	1.0		23.3	
		Yemen	1,790	9.7		38.1	
		*Subtotal*	69,731	5.8		33.7	
	Upper middle-income countries	Antigua and Barbuda	1,011	6.6		23.8	
		Argentina	1,559	2.7		24.4	
		Costa Rica	2,448	1.3		18.8	
		Dominica	1,081	1.6		23.9	
		Fiji	2,596	10.5		24.5	
		Grenada	1,091	6.6		27.4	
		Jamaica	1,378	5.9		23.5	
		Lebanon	4,183	2.5		15.6	
		Malaysia	23,185	4.5		17.4	
		Maldives	2,537	5.2		24.9	
		Mauritius	2,589	6.5		23.2	
		Namibia	2,760	9.1		43.8	
		Samoa	1,365	11.7		34.0	
		St. Lucia	1,060	6.0		25.4	
		Suriname	1,709	10.6		25.2	
		Thailand	2,194	2.7		26.7	
		*Subtotal*	52,746	3.5		22.9	
	High-income countries	Bahamas	1,100	6.9		21.4	
		Bahrain	6,314	10.6		28.0	
		Brunei Darussalam	2,316	6.3		20.6	
		Curacao	1,888	3.3		26.5	
		French Polynesia	2,506	9.9		22.9	
		Kuwait	2,606	6.4		28.0	
		Oman	2,709	4.0		41.5	
		Qatar	1,120	6.2		32.4	
		Seychelles	1,995	11.4		44.6	
		Trinidad and Tobago	2,950	7.7		14.3	
		United Arab Emirates	4,671	7.7		23.4	
		Uruguay	3,106	1.4		18.5	
		*Subtotal*	33,281	6.1		27.2	
Sex		Male	79,907	6.2	0.006	35.8	<0.001
		Female	90,711	5.2		30.8	
Age		Younger	83,496	5.9	0.313	35.3	<0.001
		Older	87,122	5.7		30.9	
Total			170,618	5.7		33.3	

### Food Insecurity (Independent Variable)

Food insecurity was inferred by the frequency of going hungry due to lack of food provision at home during the past 30 days. Options included: never, rarely, sometimes, most of the time, and always. Items very similar to the item adopted in GSHS have shown acceptable sensitivity, specificity, and reliability to detect food insecurity (41, 42). In GSHS data, each question except demographic questions and height and weight has a corresponding dichotomized variable. According to GSHS Data User's Guide (https://www.cdc.gov/gshs/pdf/gshs-data-users-guide.pdf), dichotomized variables are created by combining responses from the original question into the Response of Interest (ROI) which is the way that variables are most typically reported. Dichotomous variables are created during data processing and are the same for all GSHS data files. Their presence makes it easier to conduct comparative analyses across countries. In line with previous studies (39, 43), the dichotomous form of food insecurity were created by dividing participants into two groups: no food insecurity (combining never, rarely, and sometimes) and food insecurity (combining most of the time and always).

### Bullying Victimization (Dependent Variable)

Bullying victimization was assessed by asking participants about the number of days of being bullied during the past 30 days. The available responses varied from 0 to 30 days. In line with previous studies (44, 45), participants with a response of at least 1 day were considered bullying victims.

### Covariates

Based on the associations established in the extant literature (38, 39, 46, 47) and, more importantly, their availability in the GSHS dataset, the following variables were included as covariates; sex, age, physical fighting, current cigarette use, loneliness, number of close friends, peer support, parental connectedness, and parental bonding were considered as control variables. Specifically, to facilitate the age-stratified analysis, we classified participants into two categories: younger adolescents (aged 12–14 years) and older adolescents (aged 15–17 years) based on the common age groups used to distinguish adolescence stages (48). Items to assess these variables can be found at https://www.who.int/teams/noncommunicable-diseases/surveillance/systems-tools/global-school-based-student-health-survey/questionnaire.

### Statistical Analysis

Firstly, descriptive analyses were conducted to calculate the overall, country-stratified, sex-stratified, and age-stratified prevalence of food insecurity and bully victimization. Secondly, multivariate logistic regression was performed to investigate the country-stratified association between food insecurity and bully victimization. Higgin's *I*^2^ statistics were calculated and used to evaluate the heterogeneity between countries, where an *I*^2^ < 40% indicates negligible, and 40 to 60% represents moderate heterogeneity (49). A pooled odds ratio (OR) of the association between food insecurity and bully victimization was obtained by combining the ORs for each country into the random-effects meta-analysis. Sex, age, physical fighting, current cigarette use, loneliness, number of close friends, peer support, parental connectedness, and parental bonding were adjusted. Third, we conducted multivariate logistic regression to analyze the sex-stratified association between food insecurity and bully victimization. Country, age, physical fighting, current cigarette use, loneliness, number of close friends, peer support, parental connectedness, and parental bonding were adjusted. Fourth, to investigate the age-stratified association between food insecurity and bully victimization, we classified samples into two aged groups: younger (aged 12–14 years) and older (aged 15–17 years). Then we performed multivariate logistic regression in the age-stratified sample adjusting for country, sex, physical fighting, current cigarette use, loneliness, number of close friends, peer support, parental connectedness, and parental bonding. The results of logistic regression were presented as ORs with 95% confidence intervals (CIs). Complete case analysis was conducted prior to formal analysis. Sampling weights and the clustered sampling design of the surveys were considered in order to obtain nationally representative estimates. Control variables were adjusted for in all multivariate logistic models. Statistical significance was accepted, *a priori*, at *p* < 0.05. The above analyses were performed using SPSS 26 and Stata 16.

## Results

### Prevalence of Food Insecurity and Bullying Victimization by Countries, Sexes, and Age Groups

Table 1 provides the prevalence of each country, sex, and age group in our study. Among the 170,618 adolescents (46.8% males) included in the final sample, 48.9% belonged to the younger age groups (12–14 years) and 51.1% belonged to the older age groups (15–17 years). The pooled prevalence of food insecurity was 5.7%, varied from 3.5% in upper middle-income countries to 8.3% in low-income countries (*p* < 0.001). The prevalence of food insecurity in males was higher than that in females (males: 6.2%, females: 5.2%, *p* = 0.006). The prevalence of food insecurity was similar between younger and older adolescents (younger: 5.9%, older: 5.7%, *p* = 0.313). The pooled prevalence of bullying victimization was 33.3%, ranging from 22.9% in upper-income countries to 48.5% in low-income countries (*p* < 0.001). The prevalence of bullying victimization in males was higher than that in females (males: 35.8%, females: 30.9%, *p* < 0.001). The prevalence of bullying victimization in younger adolescents was higher than that in older ones (younger: 35.3%, older: 30.9%, *p* < 0.001).

### Overall and Country-Stratified Association Between Food Insecurity and Bully Victimization

**Figure 1:**
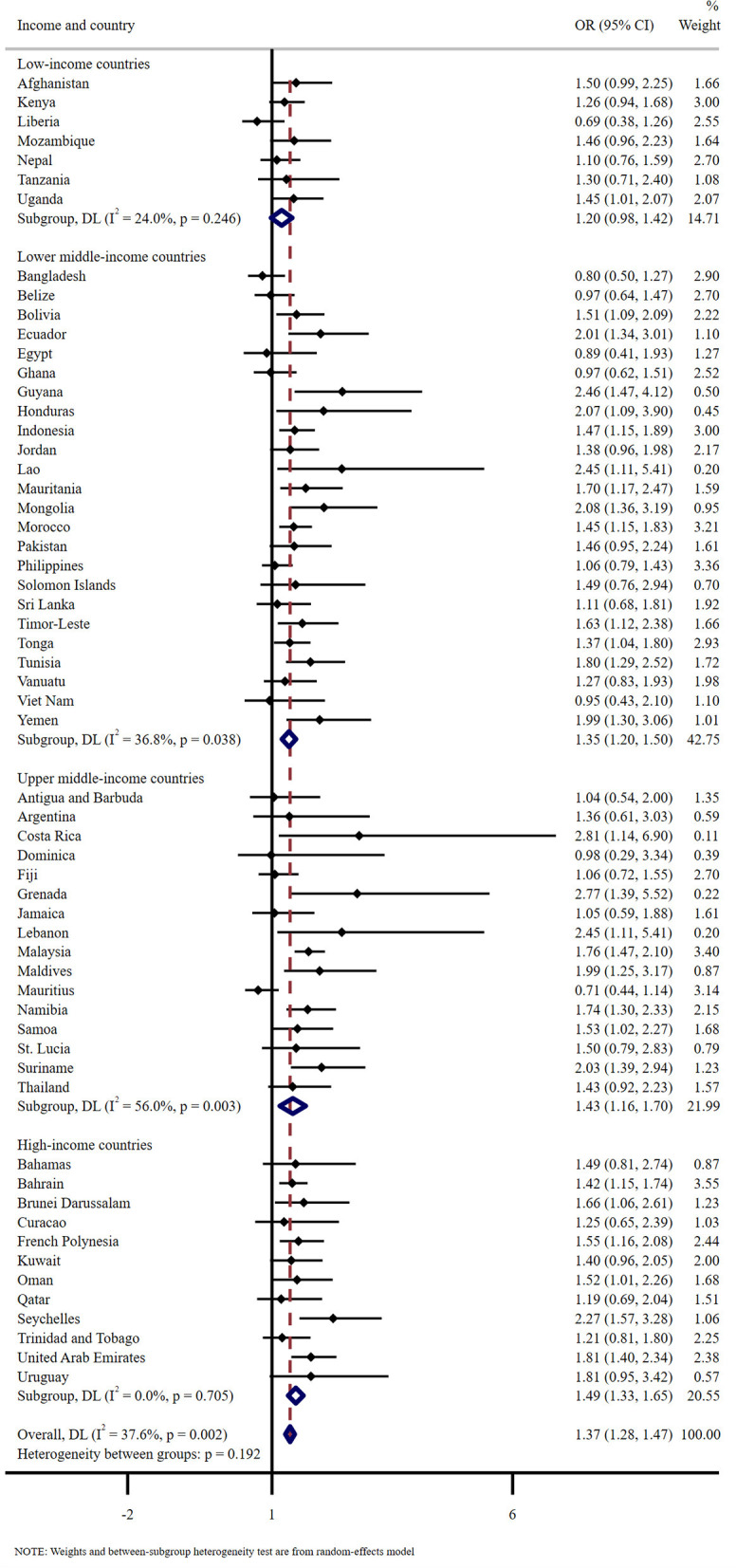
Overall and country-stratified association between food insecurity and bully victimization. The reference category is no food insecurity. Sex, age, physical fighting, current cigarette use, loneliness, number of close friends, peer support, parental connectedness, and parental bonding were adjusted. Overall estimates were obtained by meta-analysis with random effects.

Figure 1 details the results for sub-group meta-analysis according to country income levels. Overall, compared to no food insecurity, food insecurity was associated with significantly higher odds for bully victimization. The pooled OR (95% CI) was 1.37 (1.28–1.47) with negligible heterogeneity (*I*^2^ = 37.6%). Specifically, the association was stronger in higher income countries [low-income countries: OR = 1.20 (0.98, 1.42), lower middle-income countries: OR = 1.35 (1.20, 1.50), upper middle-income countries: OR = 1.43 (1.16, 1.70), high-income countries: OR = 1.49 (1.33, 1.65)].

### Sex-Stratified and Age-Stratified Association Between Food Insecurity and Bully Victimization

As Table 2 shows, the association slightly differed between sexes and age groups. Adolescents with food insecurity were more likely to experience bullying victimization in both sexes [males: OR = 1.15 (0.99, 1.34), females: OR = 1.29 (1.12, 1.49)], with stronger association observed in females. Adolescents with food insecurity also tended to experience bullying victimization in both age groups [younger: OR = 1.15 (0.99, 1.33), older: OR = 1.26 (1.10, 1.45)], and the association was more stronger in older adolescents.

**Table 2:** Sex-stratified and age-stratified association between levels of food insecurity and bullying victimization.

		**OR (95% CI)**
Sex	Male	1.15 (0.99, 1.34)^a^
	Female	1.29 (1.12, 1.49)^a^
Age	Younger	1.15 (0.99, 1.33)^b^
	Older	1.26 (1.10, 1.45)^b^

*The reference category is no food insecurity*.

a *Adjusted for country, age, physical fighting, current cigarette use, loneliness, number of close friends, peer support, parental connectedness, and parental bonding*.

b *Adjusted for country, sex, physical fighting, current cigarette use, loneliness, number of close friends, peer support, parental connectedness, and parental bonding*.

## Discussion

Using a multi-country sample derived from 170,618 adolescents in 59 countries participating in GSHS surveys, this study sought to explore the relationship between adolescent's subjective food insecurity and bullying victimization, along with the moderating effect of country income level, sex, and age. Generally, compared with adolescents without food insecurity, those with food insecurity showed significantly higher odds for experiencing bullying victimization in the total sample after adjusting for covariates. The association between food insecurity and bullying victimization was moderated by country income level, sex, and age groups.

Consistent with previous research reporting an association between food insecurity and bullying victimization among adolescents from the high-income country (USA) (36), our study further provides supporting evidence by including both relatively low-income and relatively high-income countries. Seeing food insecurity as a proxy measure of socioeconomic disadvantage or low SES (31), our findings also support a systematic review and meta-analysis indicating that the low SES may lead to higher risks of bullying victimization in adolescents (25), as well as more recent empirical studies reporting similar results (24, 36). Adolescents in low-income, food-insecure households tend to have low diet quality (50), which might cause an abnormal BMI and unhealthy body shape, leading to being too fat or too thin (51, 52). Additionally, adolescents from food-insecure households may not have enough decent clothes due to the tight household budget. Inferior physical appearance would make adolescents being alien in the school or community settings, which could result in discrimination and subsequent bullying perpetration from others (53, 54). Besides, adolescents who come from low SES families appear to have a weak sense of self-esteem (55), which may be perceived as being vulnerable or submissive that can also increase the risk for peer victimization and serves to maintain victimization (56). Generally, our finding, combined with previous literature, provide supportive evidence to the application or extension of related theories in explaining bullying involvement, especially the social capital theory (21): adolescents with low social capital (e.g., low SES), such as being food insecure, are more likely to be bullying victims than their peers.

This study also found that the association between food insecurity and bullying victimization in adolescents varied across countries with different income levels. The association was stronger in higher-income countries whereas weaker in lower-income countries, which verifies previous research on the association between food insecurity and suicide attempts (39). Food insecurity and bullying victimization were common among lower-income countries, which might attenuate the association. Besides, previous studies have shown that the individual's sense of whether he or she is better off than other people (subjective SES) is more strongly associated with mental disorders than objective indicators of SES (26, 28, 32). Other scholars have argued that socioeconomic inequality, can give rise to a sense of relative deprivation and impaired well-being (57, 58). Hence, food insecurity in countries with large socioeconomic disparities may have exaggerated impacts on adolescent's psychosocial development and thus produce a stronger association with bullying victimization. Additionally, the association was stronger among females and older adolescents. We cannot interpret the results clearly due to the limited research on similar topics. Studies have shown that females tended to be impacted more in low SES or food insecurity (59–61); the impact of food insecurity on adolescent's health outcomes varied by age groups (62, 63). Nevertheless, more research is needed to clarify the moderating effect of sex and age on the association between various SES indicators and development outcomes among adolescents.

### Limitations and Strengths

Two major study limitations must be acknowledged for a better understanding of our research findings. First, this study is a secondary data analysis and therefore cannot be replicated in the same way. Second, using data with a cross-sectional nature, which precludes causal inferences. Third, data were collected by self-reported measures to assess food insecurity and bullying victimization as well as other variables, which could increase recall bias and social desirability. Fourth, related variables such as BMI and nutrition status may provide more details to the association between food insecurity and bullying victimization, but the GSHS datasets did not include these variables. Fifth, though our data involved nearly 60 countries, we still failed to cover many countries, such as countries that are members of the Organization for Economic Cooperation and Development Economic (OECD), which limited the external validity. Future studies are encouraged to address these limitations to secure more robust evidence concerning the association between food insecurity and bullying victimization in adolescents. However, some study strengths should be mentioned. One of the strengths is the large sample (more than 170, 000) from nearly 60 countries with various income levels; so, our research findings likely have a wider range of research generalizability than many single-country studies. Second, to our knowledge, our study is one of the very few studies to assess the association between food insecurity and bullying victimization, which may increase insight into understanding bullying victimization in adolescents across the world. Third, with the large sample, we have done country-stratified, sex-stratified, and age-stratified analyses, which provided nuance information regarding the association.

### Theoretical and Practical Implications

This study further deepen our understanding of food insecurity and bullying victimization, including the impact of food insecurity and predictors of bullying victimization, which would provide implications for theory and practice. The findings of the current study are consistent with conceptual frameworks assuming individual's SES potentially serves as a strong predictor of bullying victimization (18, 21). Although theories, such as social capital theory (21), have mentioned that social status could predict bullying involvement, they stress more on bullying perpetration than bullying victimization. Our findings add supporting evidence to a more comprehensive understanding of applying these theories in bullying involvement. From a practical perspective, these findings can be applied in public health promotion and school-based bullying intervention by informing interventions to target adolescents from families at low SES. Enhancing family socioeconomic level to address adolescent's perception of food insecurity should be considered, though this action is a challenging matter and needs multiple efforts across the whole society. Considering the high prevalence of bullying victimization among adolescents, policymakers and school authorities should design and implement policies and anti-bullying interventions to address related behavioral issues. Given that partial formation of the pathway from food insecurity to bullying victimization may be attributed to the lack of social capital, some programs to increase adolescent's interpersonal competence or self-esteem could be vitalized to help strengthen in-school adolescent's school climate and social environments. This may help to mitigate the burden of bullying victimization and other related undesirable development outcomes. Moreover, owing to the association between food insecurity and bullying victimization varied across different countries, sexes, and age groups, it is extremely important to take into account the characteristics of each subgroup when conducting related interventions and research.

## Conclusion

This multi-country study highlighted that food insecurity or socioeconomic disadvantage was a correlate of bullying victimization in adolescents globally, with a stronger correlation appearing in higher-income countries, females, and older adolescents. Findings have theoretical and practical implications for understanding and addressing bullying involvement of adolescents with food insecurity or low SES.

## Data Availability Statement

Publicly available datasets were analyzed in this study. This data can be found at: https://extranet.who.int/ncdsmicrodata/index.php/catalog/GSHS.

## Ethics Statement

The studies involving human participants were reviewed and approved by all GSHS surveys, as conducted in each respective country, institutional ethical reviews. Written informed consent to participate in this study was provided by the participant's legal guardian/next of kin.

## Author Contributions

KL: formal analysis and writing-original draft. XC: writing-review and editing and project administration. S-TC: conceptualization, methodology, and writing-review and editing. CC and YZ: writing-review and editing. JW: supervision and funding acquisition. All authors contributed to and have approved the final manuscript.

## Funding

This work was supported by the Guangdong Basic and Applied Basic Research Foundation (Grant Number: 2021A1515011330), the Philosophy and Social Science Planning Project in Anhui Province (Grant Number: 2017AHSKYD3), and the Anhui University Collaborative Innovation Project (Grant Number: GXXT-2019-038).

## Conflict of Interest

The authors declare that the research was conducted in the absence of any commercial or financial relationships that could be construed as a potential conflict of interest.

## Publisher's Note

All claims expressed in this article are solely those of the authors and do not necessarily represent those of their affiliated organizations, or those of the publisher, the editors and the reviewers. Any product that may be evaluated in this article, or claim that may be made by its manufacturer, is not guaranteed or endorsed by the publisher.
